# Acute cardiovascular events after discontinuation of xanthine oxidase inhibitors: a cohort study

**DOI:** 10.1007/s10067-025-07899-7

**Published:** 2025-12-26

**Authors:** Joseph Magagnoli, Tammy H. Cummings, Meenakshi Ambati, S. Scott Sutton, Jayakrishna Ambati

**Affiliations:** 1 Department of Clinical Pharmacy and Outcomes Sciences, College of Pharmacy, University of South Carolina, Columbia, SC USA; 2 Center for Outcomes Research and Evaluation (CORE), College of Pharmacy, University of South Carolina, Coker Life Sciences 311B, 750 Sumter St., Columbia, SC 29209 USA; 3 Yale University, New Haven, CT USA; 4 Center for Advanced Vision Science, University of Virginia School of Medicine, Charlottesville, VA USA; 5 Department of Ophthalmology, University of Virginia School of Medicine, Charlottesville, VA USA; 6 Department of Pathology, University of Virginia School of Medicine, Charlottesville, VA USA; 7 Department of Microbiology, Immunology, and Cancer Biology, University of Virginia School of Medicine, Charlottesville, VA USA

**Keywords:** Allopurinol, Cardiovascular events, Discontinuation, Febuxostat, Xanthine oxidase inhibitors

## Abstract

**Background:**

Xanthine oxidase inhibitors (XOis) are commonly used to treat gout and hyperuricemia. Beyond urate-lowering effects, XOis may influence cardiovascular outcomes via oxidative stress pathways. Prior evidence, including post hoc analyses of the CARES trial, suggests increased mortality after XOi discontinuation, raising concern for a potential “withdrawal syndrome.” However, evidence from real-world outpatient populations is limited.

**Objective:**

This study aims to evaluate whether the recent discontinuation of XOi therapy is associated with an increased risk of acute cardiovascular events in patients with gout.

**Methods:**

We conducted a retrospective cohort study using the Merative MarketScan database. Adults with gout initiating allopurinol or febuxostat were included. Discontinuation was defined as no XOi supply in the prior 90 days during the 121- to 180-day window post-initiation. The primary outcome was hospitalization or outpatient diagnosis of acute myocardial infarction or ischemic stroke. Cox proportional hazards models with stabilized inverse probability weights were used to estimate hazard ratios (HRs), adjusting for demographic and clinical covariates.

**Results:**

Among 508,872 patients initiating XOi therapy, 23.6% discontinued therapy within the first 121- to 180-day post-initiation timeframe. Discontinuers were younger with fewer comorbidities at baseline. After weighting, groups were well balanced. XOi discontinuation was associated with a modest but statistically significant increased risk of acute cardiovascular events (HR, 1.05; 95% CI, 1.01–1.09; *p* = 0.019). The magnitude of the effect increases among patients with preexisting hypertension diagnoses (HR, 1.13; 95% CI, 1.03–1.23; *p* = 0.006).

**Conclusions:**

In this large real-world cohort, early discontinuation of XOi therapy was linked to a small but significant elevation in cardiovascular risk. These findings support prior signals of potential harm from XOi withdrawal, particularly among patients with cardiovascular disease, and highlight the importance of sustained therapy adherence.

**Table:** 

**Key Points** • *Xanthine oxidase inhibitor (XOi) discontinuation was associated with a modest but significant increase in acute cardiovascular events in a large national cohort of patients with gout.* •* Even early discontinuation after XOi initiation may increase cardiovascular risk, underscoring the importance of treatment persistence.* • *Adherence to XOi therapy may be an important factor in reducing cardiovascular risk among gout patients.*

**Supplementary Information:**

The online version contains supplementary material available at 10.1007/s10067-025-07899-7.

## Introduction

Xanthine oxidase inhibitors (XOis), including allopurinol and febuxostat, are the mainstay of rate-lowering therapy for gout and hyperuricemia [1]. In addition to their role in controlling serum uric acid, XOis may influence cardiovascular risk through modulation of oxidative stress and endothelial function [2–4]. The Cardiovascular Safety of Febuxostat and Allopurinol in Participants with Gout and Cardiovascular Morbidities (CARES) trial found that febuxostat was associated with a higher risk of all-cause and cardiovascular mortality compared with allopurinol among patients with established cardiovascular disease [5]. Notably, post hoc analyses revealed that much of the excess mortality occurred when patients were no longer receiving either study XOi [6, 7], raising the possibility of a xanthine oxidase inhibitor withdrawal syndrome [8].

Mechanistically, xanthine oxidase catalyzes the oxidation of hypoxanthine to xanthine and subsequently to uric acid (UA). At physiological concentrations, UA may exert antioxidant effects, but at elevated levels, it can function as a pro-oxidant, contributing to the generation of reactive oxygen species (ROS), endothelial dysfunction, and inflammation [9]. Suppression of xanthine oxidase activity through chronic XOi therapy could therefore attenuate oxidative stress, while withdrawal might reverse these effects, resulting in increased cardiovascular risk. Supporting this hypothesis, a systematic review reported that XOi use was associated with a modest degree of cardiovascular protection [10], and an inpatient cohort study observed increased mortality following XOi discontinuation in high-risk inpatients [11].

Despite these signals, evidence regarding the cardiovascular consequences of XOi withdrawal in real-world outpatient populations remains limited. To our knowledge, no large-scale study has evaluated the association between XOi discontinuation and the risk of acute cardiovascular events. In the present study, we use a large national claims database to test the hypothesis that recent discontinuation of XOi therapy is associated with an increased risk of acute cardiac diagnoses, including acute myocardial infarction and ischemic stroke.

## Materials and methods

### Data source

We performed a retrospective cohort study using the Merative MarketScan Commercial Claims and Encounters and Medicare Supplemental databases. These databases contain inpatient, outpatient, and pharmacy claims for individuals with employer-sponsored health insurance and Medicare-eligible retirees with supplemental coverage, including service dates, diagnosis and procedure codes, prescription dispensations, and enrollment records.

### Study cohort

Adults with a diagnosis of gout were identified from inpatient or outpatient claims using ICD-9-CM codes 274.x or ICD-10-CM code M10.xx. The gout index date was defined as the earliest qualifying diagnosis. Initiation of a xanthine oxidase inhibitor (XOi), either allopurinol or febuxostat, was identified from outpatient pharmacy claims using National Drug Codes (NDC). The XOi index date was the earliest dispensing date with a days’ supply greater than zero, occurring on or after the gout index date.

### Exposure and outcome

**Fig. 1:**
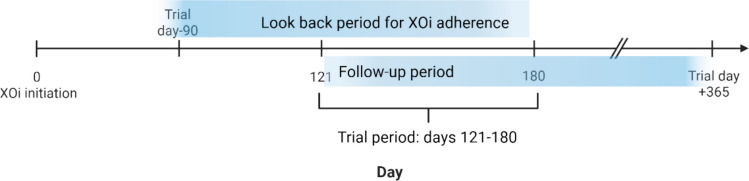
Study design for time-varying exposure analysis of xanthine oxidase inhibitor (XOi) discontinuation (created with Biorender.com). For each eligible trial day between days 121 and 180 after XOI initiation, exposure status was classified based on the preceding 90 days: any supply of allopurinol or febuxostat = continued use and no supply = discontinued. Follow-up for each trial day began on that trial day and continued until the earliest next trial day, acute cardiovascular event, end of eligibility, 365 days post-trial day, or study. This design treats discontinuation status as a time-varying exposure and avoids immortal time bias by ensuring exposure classification is based solely on pre-follow-up information for each trial day

The primary exposure was XOi discontinuation, defined as having no allopurinol or febuxostat supply in the 90 days preceding each study day (between days 121 and 180 after XOi initiation) (Fig. 1). Days of drug exposure were derived by expanding each dispensing to cover its recorded days’ supply. Discontinuation status was assessed dynamically for each eligible day during the study period. The primary outcome was an acute cardiovascular event, defined as hospitalization or outpatient visit with a diagnosis of acute myocardial infarction (AMI) or ischemic stroke. Events were identified using ICD-9-CM codes 410.x, 411.x, and 433.x–436.x and ICD-10-CM codes I21.x, I22.x, I24.x, I63.x, I65.x, and I66.x. The earliest qualifying event date was used; patients with any acute cardiovascular event prior to XOi initiation were excluded. Patients are often initiated on colchicine as prophylaxis for gout flares when starting XOi therapy. Colchicine could also be cardioprotective. To address the possibility that the observed association reflected simultaneous cessation of colchicine rather than XOi therapy, we incorporated colchicine use (within the past 90 days) as a time-varying covariate in all models.

### Covariates

Baseline covariates included age, sex, year of XOi initiation, Charlson Comorbidity Index score, and comorbidity indicators for pure hypercholesterolemia, hypertriglyceridemia, hyperlipidemia, other heart disease, hypertension, type 2 diabetes mellitus (T2DM), depression, alcohol dependence, smoking, and obesity. Comorbidities were identified using ICD-9-CM and ICD-10-CM codes from all available inpatient and outpatient claims prior to trial entry. ICD-9/10-CM codes are listed in Supplemental Table S1.

### Statistical analysis

Baseline characteristics were summarized using means and standard deviations for continuous variables and counts with percentages for categorical variables. Comparisons between discontinuation statuses were performed using *t*-tests for continuous variables and *χ*^2^ tests for categorical variables. Standardized mean differences (SMDs) were used to assess covariate balance between groups, with balance evaluated before and after application of the study weights; weighted SMDs < 0.1 were considered indicative of adequate balance.

The primary exposure, XOi discontinuation, was evaluated in a time-varying framework, at each study day between days 121 and 180 after XOi initiation. Follow-up for each study day continued until the earliest occurrence of the next trial day, an acute cardiovascular event, loss of eligibility, or 365 days after trial entry.

We fit Cox proportional hazards models to estimate hazard ratios (HRs) and 95% confidence intervals (CIs) for the association between XOi discontinuation in the prior 90 days and incident acute cardiovascular events. Models were stratified by day (121–180) to allow for varying baseline hazards and clustered on patients to account for repeated measures per patient. All models adjusted for age, sex, year of XOi initiation, Charlson Comorbidity Index score, and comorbidity indicators for pure hypercholesterolemia, hypertriglyceridemia, hyperlipidemia, other heart disease, hypertension, type 2 diabetes mellitus, depression, alcohol dependence, smoking, and obesity.

To address potential confounding and artificial censoring, we applied stabilized inverse probability weights. Inverse probability of treatment weights (IPTW) were estimated from a pooled logistic regression model for XOi discontinuation that included all baseline covariates and natural spline terms for trial day. Inverse probability of censoring weights (IPCW) were estimated from pooled logistic models for remaining eligible, with the denominator model including baseline covariates and spline terms for study day and the numerator model including spline terms for trial day only. The final weight for each observation was the product of IPTW and IPCW. Stabilized weights were truncated at the 1 st and 99th percentiles to reduce the influence of extreme values. The weighted Cox model used the same covariates, stratification by trial day, and clustering as in the unweighted model. All analyses were conducted in R (R Foundation for Statistical Computing, Vienna, Austria). Two-sided *p*-values < 0.05 were considered statistically significant.

## Results

A total of 508,872 patients with gout who initiated XOi therapy (allopurinol or febuxostat) were included in the analytic cohort. The mean (SD) age was 54.1 (13.0) years, and 83.2% were male (Table 1). Almost 24% of patients were dispensed colchicine along with their initial XOi therapy. The mean Charlson Comorbidity Index score was 0.87 (1.57). Common baseline comorbidities included hypertension (52.7%), hyperlipidemia (26.9%), type 2 diabetes mellitus (18.7%), pure hypercholesterolemia (10.5%), and hypertriglyceridemia (10.8%). Other heart disease was present in 11.2% of patients, while depression, alcohol dependence, smoking, and obesity were identified in 4.2%, 0.7%, 4.5%, and 11.5%, respectively. The mean (SD) year of XOi initiation was 2013.36 (4.6).

**Table 1:** Sample characteristics

Variable	Level	Overall sample	Any discontinuation		*p*-value	Standardized difference
		*N* = 508,872	No (*N* = 388,656)	Yes (*N* = 120,216)		
Age	Baseline	54.1 (13.03)	55.02 (12.89)	51.14 (13.04)	< 0.001	0.3
Sex	Male	423,266 (83.2%)	322,756 (83%)	100,510 (83.6%)	< 0.001	0.02
	Female	85,606 (16.8%)	65,900 (17%)	19,706 (16.4%)	< 0.001	0.02
Charlson comorbidity index		0.87 (1.57)	0.91 (1.61)	0.71 (1.45)	< 0.001	0.13
Pure hypercholesterolemia		53,408 (10.5%)	42,563 (11%)	10,845 (9%)	< 0.001	0.06
Hypertriglyceridemia		54,896 (10.8%)	44,332 (11.4%)	10,564 (8.8%)	< 0.001	0.09
Hyperlipidemia		136,801 (26.9%)	109,522 (28.2%)	27,279 (22.7%)	< 0.001	0.13
Other heart disease		57,226 (11.2%)	45,715 (11.8%)	11,511 (9.6%)	< 0.001	0.07
Hypertension		268,335 (52.7%)	213,085 (54.8%)	55,250 (46%)	< 0.001	0.18
T2DM		95,335 (18.7%)	77,054 (19.8%)	18,281 (15.2%)	< 0.001	0.12
Depression		21,243 (4.2%)	16,422 (4.2%)	4821 (4%)	0.001	0.01
Alcohol dependence		3348 (0.7%)	2425 (0.6%)	923 (0.8%)	< 0.001	0.02
Smoking		22,987 (4.5%)	17,165 (4.4%)	5822 (4.8%)	< 0.001	0.02
Obesity		58,406 (11.5%)	46,327 (11.9%)	12,079 (10%)	< 0.001	0.06
Colchicine co-prescribed		120,587 (23.7%)	90,889 (23.4%)	29,698 (24.7%)	< 0.001	0.03
XOi medication						
Allopurinol		478,457 (94.02%)	365,909 (94.1%)	112,548 (93.6%)	< 0.001	0.02
Febuxostat		30,415 (5.98%)	22,747 (5.9%)	7668 (6.4%)	< 0.001	0.02
Year XOi initiation		2013.36 (4.6)	2013.58 (4.65)	2012.64 (4.35)	< 0.001	0.21

When classified into any discontinuation between days 121 and 180 after initiation, 120,216 patients (23.6%) met the definition for discontinuation, while 388,656 (76.4%) did not. Patients who discontinued tended to be younger (mean age 51.1 vs 55.0 years; standardized difference 0.30), with lower comorbidity burden (Charlson score, 0.71 vs 0.91; standardized difference, 0.13). Discontinuers also had lower prevalence of hypertension (46.0% vs 54.8%; standardized difference 0.18), type 2 diabetes mellitus (15.2% vs 19.8%; standardized difference 0.12), and hyperlipidemia (22.7% vs 28.2%; standardized difference 0.13). Year of XOi initiation differed slightly between groups (2012.6 vs 2013.6; standardized difference 0.21), with discontinuers initiating slightly earlier on average. There was minimal difference in terms of colchicine prescribing with initial XOi between groups, with those with a discontinuation having had colchicine co-prescribed at 24.7% and those who did not discontinue at 23.4%. Other comorbidities, including depression, alcohol dependence, and smoking, showed small absolute differences (standardized differences ≤ 0.02). After applying stabilized inverse probability of treatment and censoring weights, baseline characteristics were well balanced between discontinuers and continuers. All standardized mean differences were < 0.1, indicating negligible residual imbalance at select trial days 121, 150, and 180 (Supplemental Table S1).

Table 2 shows the results of the primary analysis model. During follow-up, recent XOi discontinuation in the prior 90 days was associated with a modest but statistically significant increase in the hazard of acute cardiovascular events (unweighted HR, 1.06; 95% CI, 1.02–1.11; *p* = 0.002). This association persisted after the application of stabilized inverse probability weights (IPTW HR, 1.05; 95% CI, 1.01–1.09; *p* = 0.019). Colchicine use within the prior 90 days demonstrated a small positive association with acute cardiovascular events (unweighted HR, 1.04; 95% CI, 1.00–1.08), with a similar estimate observed in the weighted model (IPTW HR, 1.04; 95% CI, 0.99–1.08). Several baseline factors—including older age, higher Charlson Comorbidity Index score, pure hypercholesterolemia, hypertriglyceridemia, other heart disease, hypertension, type 2 diabetes mellitus, depression, alcohol dependence, and smoking—were independently associated with increased cardiovascular risk in the IPTW-adjusted model. More recent years of XOi initiation were associated with a lower hazard of events.

**Table 2:** Risk of acute cardiovascular event: Cox model

Predictor	Original		IPTW	
	HR (95% CI)	*p*-value	HR (95% CI)	*p*-value
No XOi last 90 days	1.06 (1.02–1.11)	0.002	1.05 (1.01–1.09)	0.019
Age	1.05 (1.05–1.05)	< 0.001	1.05 (1.05–1.05)	< 0.001
Female	0.96 (0.93–1)	0.042	0.97 (0.93–1.01)	0.09
Charlson comorbidity	1.09 (1.09–1.1)	< 0.001	1.09 (1.08–1.1)	< 0.001
Pure hypercholesterolemia	1.1 (1.05–1.14)	< 0.001	1.1 (1.05–1.15)	< 0.001
Hypertriglyceridemia	1.11 (1.05–1.16)	< 0.001	1.1 (1.04–1.15)	< 0.001
Hyperlipidemia	1.03 (0.99–1.06)	0.121	1.02 (0.99–1.06)	0.219
Other heart disease	1.51 (1.46–1.57)	< 0.001	1.53 (1.47–1.59)	< 0.001
Hypertension	1.12 (1.08–1.16)	< 0.001	1.13 (1.09–1.17)	< 0.001
T2DM	1.17 (1.13–1.22)	< 0.001	1.17 (1.13–1.22)	< 0.001
Depression	1.22 (1.14–1.31)	< 0.001	1.22 (1.13–1.31)	< 0.001
Alcohol dependence	1.34 (1.13–1.6)	0.001	1.34 (1.11–1.6)	0.002
Smoking	1.17 (1.1–1.26)	< 0.001	1.16 (1.08–1.25)	< 0.001
Obesity	0.96 (0.91–1.01)	0.145	0.97 (0.92–1.02)	0.232
XOi initiation year	0.95 (0.94–0.95)	< 0.001	0.95 (0.95–0.95)	< 0.001
Colchicine use within last 90 days	1.04 (1–1.08)	0.03	1.04 (0.99–1.08)	0.063

Table 3 shows the results of the interactive model, fit with interactions between hypertension and XOi discontinuation as well as hypertension with colchicine use. The interaction model revealed that the association between recent XOi discontinuation and cardiovascular risk differed by hypertension status. Among patients without hypertension, discontinuation was not associated with increased cardiovascular risk (IPTW HR, 0.97; 95% CI, 0.90–1.04). However, the interaction term showed that discontinuation was associated with a higher hazard of acute cardiovascular events among individuals with hypertension (interaction IPTW HR, 1.13; 95% CI, 1.03–1.23). In addition, the colchicine × hypertension interaction term yielded point estimates below 1.0, suggesting that colchicine use may attenuate cardiovascular risk among hypertensive patients in both unweighted and weighted analyses (IPTW HR, 0.93; 95% CI, 0.86–1.01). However, the colchicine and hypertension interaction term was not statistically significant in either model.

**Table 3:** Interaction model

Predictor	Original		IPTW	
	HR (95% CI)	*p*-value	HR (95% CI)	*p*-value
No XOi last 90 days	0.97 (0.9–1.03)	0.314	0.97 (0.9–1.04)	0.319
Hypertension	1.1 (1.06–1.15)	< 0.001	1.12 (1.07–1.17)	< 0.001
No XOi last 90 days × hypertension	1.16 (1.07–1.26)	< 0.001	1.13 (1.03–1.23)	0.006
Age	1.05 (1.05–1.05)	< 0.001	1.05 (1.05–1.05)	< 0.001
Female	0.96 (0.93–1)	0.041	0.97 (0.93–1.01)	0.09
Charlson comorbidity	1.1 (1.09–1.1)	< 0.001	1.09 (1.08–1.1)	< 0.001
Pure hypercholesterolemia	1.1 (1.05–1.14)	< 0.001	1.1 (1.05–1.15)	< 0.001
Hypertriglyceridemia	1.11 (1.06–1.16)	< 0.001	1.1 (1.04–1.15)	< 0.001
Hyperlipidemia	1.03 (0.99–1.06)	0.118	1.02 (0.99–1.06)	0.222
Other heart disease	1.51 (1.46–1.57)	< 0.001	1.53 (1.47–1.59)	< 0.001
T2DM	1.17 (1.13–1.22)	< 0.001	1.17 (1.13–1.22)	< 0.001
Depression	1.22 (1.14–1.31)	< 0.001	1.22 (1.13–1.31)	< 0.001
Alcohol dependence	1.34 (1.12–1.59)	0.001	1.34 (1.11–1.6)	0.002
Smoking	1.17 (1.1–1.26)	< 0.001	1.16 (1.08–1.25)	< 0.001
Obesity	0.96 (0.91–1.01)	0.15	0.97 (0.92–1.02)	0.239
XOi initiation year	0.95 (0.94–0.95)	< 0.001	0.95 (0.95–0.95)	< 0.001
Colchicine use within last 90 days	1.09 (1.02–1.16)	0.011	1.09 (1.02–1.17)	0.011
Colchicine use within last 90 days × hypertension	0.94 (0.86–1.01)	0.109	0.93 (0.86–1.01)	0.076

## Discussion

A post-hoc analysis of the CARES trial [5] found that much of the excess mortality occurred after patients discontinued their study XOi [6, 7], which suggests a possible xanthine oxidase inhibitor withdrawal syndrome [8]. Uric acid (UA) can act as an antioxidant, but at high levels, it can also function as a pro-oxidant, contributing to the generation of reactive oxygen species (ROS), endothelial dysfunction, and inflammation [9]. This suggests that chronic XOi therapy might reduce oxidative stress, while stopping the treatment could reverse these effects and increase cardiovascular risk. Previous research supports this idea; a systematic review showed that XOi use was associated with a modest degree of cardiovascular protection [10], and a study of hospitalized patients observed higher mortality after XOi was discontinued in high-risk individuals [11].

In this large real-world cohort, we found that discontinuation of XOi therapy between days 121 and 180 after initiation was associated with a modest but statistically significant increase in the risk of acute cardiovascular events over the following year (HR, 1.05). The consistency of estimates across unweighted and weighted models strengthens the inference that interruption or cessation of therapy may increase cardiovascular risk. Although the absolute effect size was small, the finding is clinically meaningful given the high prevalence of gout and the common occurrence of early discontinuation in routine care. Importantly, our interaction analyses suggest that the elevated cardiovascular risk associated with discontinuation may be concentrated among patients with hypertension. Among individuals without hypertension, discontinuation was not associated with increased risk, whereas hypertensive patients experienced a significantly higher hazard of acute cardiovascular events when XOi therapy was stopped. This pattern indicates that XOi withdrawal may be particularly deleterious in individuals with existing cardiovascular risk factors, a finding consistent with the biological premise that these patients may be more susceptible to abrupt changes in oxidative stress or endothelial function. The interaction model therefore supports the notion that the cardiovascular consequences of discontinuation are not uniform across patients but instead may be amplified among those already predisposed to vascular events.

This subgroup finding also aligns directionally with the CARES trial, which enrolled patients with established cardiovascular disease, a population inherently at higher baseline risk. In CARES, the excess mortality after treatment discontinuation occurred in a group with substantial cardiorenal comorbidity, and our results suggest that a similar vulnerability may extend to hypertensive patients in real-world settings. Taken together, these findings reinforce the possibility that XOi withdrawal has the greatest impact among patients with preexisting cardiovascular risk, providing a potential explanation for the stronger signal observed in CARES compared with the more modest effect observed in our broader gout population. Given the heterogeneous phenotypes of gout patients, such as those identified by Richette et al. [12, 13], more analysis to specify the cardiovascular risk of XOi discontinuation is needed.

In addition to the differential effect observed with hypertension, our interaction analyses also suggested that colchicine use may modify cardiovascular risk among patients at higher baseline risk. Although the colchicine × hypertension interaction did not meet conventional thresholds for statistical significance, the point estimates were consistently below 1.0, indicating a possible attenuation of cardiovascular risk among hypertensive individuals receiving colchicine. This directional signal is notable given the growing body of evidence supporting the cardioprotective effects of low-dose colchicine through suppression of NLRP3-mediated inflammation and reduction of vascular inflammatory activity [14]. While our study was not designed to evaluate colchicine as a primary cardiovascular therapy, the pattern observed in the interaction model suggests that colchicine use may partially mitigate the adverse cardiovascular consequences of XOi discontinuation in high-risk patients. These findings warrant further investigation, particularly considering emerging data supporting colchicine for cardiovascular prevention [15–17].

Strengths of our analysis include the large sample size, detailed longitudinal prescription data, and use of weighting to reduce confounding and artificial censoring. Nonetheless, limitations remain. First, residual confounding by unmeasured factors (serum urate levels, renal function) is possible. Second, discontinuation was defined as having 0 days of XOI supply in the 90 days prior to a trial day, whereas patients with even a single day of supply in that window were classified as continuers. This strict definition may have led to misclassification of exposure, particularly for patients who used medication intermittently, had leftover supply from earlier fills, or had just refilled after a gap but with minimal coverage. Third, mortality outcomes were unavailable, limiting direct comparison with the CARES trial. Finally, the HR observed, while statistically significant, was modest overall; future studies warrant the investigation of patients in the highest risk categories. Fourth, we did not compare the effects of discontinuing allopurinol versus febuxostat. Allopurinol accounted for approximately 94% of XOI prescriptions in our cohort, and therefore our findings primarily reflect discontinuation of allopurinol. Future studies with larger febuxostat-treated populations are needed to determine whether the cardiovascular consequences of discontinuation differ by agent or dose, particularly given febuxostat’s greater potency as a xanthine oxidase inhibitor. In addition, we were unable to ascertain the clinical reason for discontinuation, including whether cessation was related to adverse effects, changes in disease severity, or patient preference. Prior research indicates that discontinuation of gout medications is common, with fewer than 50% of patients remaining adherent to therapy at one year—and in some studies, adherence rates as low as 35% [18, 19], driven by diverse factors such as lifestyle considerations, perceived lack of need, and treatment inconvenience, rather than side effects alone [20].

Despite these limitations, our findings demonstrate that the discontinuation of XOi therapy in routine clinical practice may have cardiovascular implications, particularly among patients with underlying cardiovascular risk factors such as hypertension. These results highlight the potential importance of sustained XOi therapy in reducing cardiovascular vulnerability among higher-risk individuals.

## Supplementary Information

Below is the link to the electronic supplementary material.

ESM 1 Supplementary Material 1 (DOCX 17.4 KB)

## Data Availability

Data are available through agreements with Merative, the owners of MarketScan.
