# Supplementary material for: Acute cardiovascular events after discontinuation of xanthine oxidase inhibitors: a cohort study
Source: Clin Rheumatol. 2025 Dec 26;45(2):1431–8. doi: 10.1007/s10067-025-07899-7 (PMC12858605; doi:10.1007/s10067-025-07899-7)
Supplement: Supplementary file 1 — Supplementary Material 1 (DOCX 17.4 KB) [file 10067_2025_7899_MOESM1_ESM.docx]

**Supplemental Table S1: Diagnosis Codes for Baseline Covariates**

| **Covariate** | **ICD-9-CM Codes** | **ICD-10-CM Codes** |
| --- | --- | --- |
| **Pure hypercholesterolemia** | 2720 | E7800 |
| **Hypertriglyceridemia** | 2721, 2722 | E781, E782 |
| **Hyperlipidemia** | 2723, 2724 | E783, E784, E785 |
| **Other heart disease** | 420, 421, 422, 424, 4240, 4241, 4242, 4243, 425, 4250, 4252, 4253, 4254, 4255, 4257, 4258, 4259, 426, 4260, 42611, 42612, 42613, 4263, 4264, 4266, 4267, 427, 4270, 4273, 42731, 4274, 42741, 4275, 4276, 4278, 42781, 42789, 4279, 428, 4280, 4281, 4282, 4283, 4284, 429, 4290, 4291, 4292, 4293, 4294, 4295, 4296, 4297, 42971, 42979, 4298, 42981, 42982, 42983, 42989, 4299 | I30, I31, I310, I311, I312, I313, I318, I319, I33, I34, I340, I341, I342, I35, I350, I351, I352, I36, I360, I361, I362, I370, I371, I372, I38, I39, I50, I500, I501, I509, I51, I510, I511, I512, I513, I514, I515, I516, I517, I518, I519 |
| **Hypertension** | 401, 402, 403, 404, 405 | I10, I11, I12, I13, I15 |
| **Type 2 diabetes mellitus (T2DM)** | 25000, 25002, 25010, 25012, 25020, 25022, 25030, 25032, 25040, 25042, 25050, 25052, 25060, 25062, 25070, 25072, 25080, 25082, 25090, 25092 | E11 |
| **Depression** | 311, 29621–29626, 29682, 2963 | F32, F33 |
| **Alcohol dependence** | 3039 | F102 |
| **Smoking** | 3051, V1582 | Z720, Z87891, F17210 |
| **Obesity** | 278 | E66 |

Supplemental Table S2. Weighted standardized differences

| Variable | Standardized difference | |  |
| --- | --- | --- | --- |
|  | Day 121 | Day 150 | Day 180 |
| Age | 0.0268 | 0.0116 | 0.0096 |
| Sex | 0.0005 | 0.0019 | 0.0006 |
| Charlson comorbidity index | 0.0171 | 0.0071 | 0.0015 |
| Pure hypercholesterolemia | 0.0002 | 0.0002 | 0.0009 |
| Hypertriglyceridemia | 0.0035 | 0.0013 | 0.001 |
| Hyperlipidemia | 0.0033 | 0.0026 | 0.0008 |
| Other heart disease | 0.0023 | 0.0002 | 0.0019 |
| Hypertension | 0.005 | 0.0014 | 0.0008 |
| T2DM | 0.0024 | 0.001 | 0.0016 |
| Depression | 0.0002 | 0.0007 | 0.0002 |
| Alcohol dependence | 0.0001 | 0.0001 | 0.0002 |
| Smoking | 0 | 0.0001 | 0.0005 |
| Obesity | 0.0038 | 0.0018 | 0.0007 |
| Year XOi initiation | 0.0403 | 0.0215 | 0.015 |
| Colchicine use last 90 days | 0.0087 | 0.0168 | 0.0089 |
